# Characteristics and functions of volatile organic compounds in the tripartite symbiotic system of *Gastrodia elata-Armillaria gallica-Rahnella aceris* HPDA25

**DOI:** 10.1080/15592324.2024.2399426

**Published:** 2024-09-04

**Authors:** Ying Zhang, Tianrui Liu, Tiegui Nan, Zhongyi Hua, Yuyang Zhao, Yuan Yuan

**Affiliations:** a State Key Laboratory for Quality Ensurance and Sustainable Use of Dao-di Herbs, National Resource Center for Chinese Materia Medica, China Academy of Chinese Medical Sciences, Beijing, China; b Jiangxi Province Key Laboratory of Sustainable Utilization of Traditional Chinese Medicine Resources, Institute of Traditional Chinese Medicine Health Industry, China Academy of Chinese Medical Sciences, Nanchang, China; c Jiangxi Health Industry Institute of Traditional Chinese Medicine, Nanchang, China; d Experimental Research Center, China Academy of Traditional Chinese Medicine, Beijing, China

**Keywords:** Orchid mycorrhizal symbiosis, volatile organic compounds, plant growth-promoting bacteria, *Gastrodia elata*, *Armillaria*

## Abstract

Tripartite interactions among plants, fungi, and bacteria are critical for maintaining plant growth and fitness, and volatile organic compounds (VOCs) play a significant role in these interactions. However, the functions of VOCs within the niche of mycoheterotrophic plants, which represent unique types of interactions, remain poorly understood. *Gastrodia elata*, a mycoheterotrophic orchid species, forms a symbiotic relationship with specific *Armillaria* species, serving as a model system to investigate this intriguing issue. *Rahnella aceris* HPDA25 is a plant growth-promoting bacteria isolated from *G. elata*, which has been found to facilitate the establishment of *G. elata-Armillaria* symbiosis. In this study, using the tripartite symbiotic system of *G. elata-Armillaria gallica-R. aceris* HPDA25, we investigate the role of VOCs in the interaction among mycoheterotrophic plants, fungi, and bacteria. Our results showed that 33 VOCs of HPDA25-inducible symbiotic *G. elata* elevated compared to non-symbiotic *G. elata*, indicating that VOCs indeed play a role in the symbiotic process. Among these, 21 VOCs were accessible, and six active VOCs showed complete growth inhibition activities against *A. gallica*, while *R. aceris* HPDA25 had no significant effect. In addition, three key genes of *G. elata* have been identified that may contribute to the increased concentration of six active VOCs. These results revealed for the first time the VOCs profile of *G. elata* and demonstrated its regulatory role in the tripartite symbiotic system involving *G. elata*, *Armillaria*, and bacteria.

## Introduction

The *Armillaria* from Basidiomycota is a pathogen that causes destructive root white rot disease in various tree species.^1^ *Armillaria* species grow by colonizing living root and, after killing the root cambium, harnessing nutrients from dead tissues.^1,2^ Plant cell wall-degrading enzymes (PCWDEs) play vital roles in this process.^3^ PCWDEs are a diverse group of enzymes that catalyze the breakdown of the complex carbohydrates composing plant cell walls.^4^ Plant cell walls are primarily composed of cellulose, hemicellulose, and pectin.^5^ Consequently, the extent to which *Armillaria* breaches plant defenses and colonizes tissues can be characterized by the activities of enzymes such as laccase, cellulase, xylanase, pectinase, and amylase.

Although *Armillaria* is a notable pathogen, *Gastrodia elata*, a mycoheterotrophic orchid species, can form a symbiotic relationship with particular *Armillaria* species.^6^ *G. elata* is a fully mycoheterotrophic plant, relying entirely on mycorrhizal fungi for its carbon and energy needs, having lost its photosynthesis through evolution.^7^ During the early stages of its life cycle, *G. elata* tubers associate closely with the fungal hyphae, extracting essential nutrients from the fungal mycelium to support its growth and development.^1^ Although there are around 400 fully mycoheterotrophic plant species in 87 genera and 10 families, only *G. elata* relies on *Armillaria*.^8^ This distinctive symbiotic relationship is the only known example of a plant harnessing *Armillaria* to obtain essential nutrients, thereby playing a crucial role in managing fungi diseases and investigating plant–fungus interactions. The mechanisms underlying this specific symbiosis, particularly the defensive capabilities of *G. elata* against *Armillaria*, have been investigated from various perspectives. The *Gastrodia* anti-fungal protein (GAFP; gastrodianin) was the most widely studied. More than 80% of the GAFP genes in *G. elata* exhibit high transcript levels during the growth stage prior to establishing a stable symbiotic relationship with *Armillaria*.^7^ Phytohormones are another important factor in the *G. elata-Armillaria* symbiosis. Auxins have been reported to mediate the expression of *Armillaria* genes involved in regulating cell growth and metabolism,^9^ and strigolactone may also impact *A. gallica* growth by modulating the reactive oxygen species.^10^

However, apart from phytohormones, the impact of other small compounds from *G. elata* on *Armillaria* has not been explored enough. Volatile organic compounds (VOCs) are a wide range of metabolites released by plants throughout their life cycle^11^ and have diverse effects on plants,^12,13^ fungi,^14^ and bacteria,^15^ thereby attracting increased interest in research of plant–microbe interactions. Plant–plant interactions have focused on the inducible defense of plant under biotic and abiotic stress, e.g., tomato plants infected with *Bemisia tabaci* have been observed to release VOCs, which enhance defenses against pathogens in neighboring plants.^16^ In addition to inducible direct resistance, VOCs mediate indirect plant defenses against plant pathogens by inhibiting pathogen fungi,^17^ e.g., methyl propanoate and methyl prop-2-enoate from barley significantly inhibit the growth and spore germination of *Fusarium culmorum* and *Cochliobolus sativus*.^18^ VOCs also regulate rhizosphere bacteria,^19^ which also play a crucial role in *G. elata* health,^20^ and the VOC methyl jasmonate (MeJA) derived from roots positively affect beneficial bacteria in the soil.^15^

Although the effects of VOCs on plants, fungi, and bacteria have been preliminarily elucidated separately, comprehensive research that investigate plant-fungi-bacteria interactions holistically is still lacking. Unlike other plant–pathogen interactions, the relationship between *G. elata* and *Armillaria* develops spontaneously, thus offering greater research potential. In our recent study, a rhizosphere bacteria *Rahnella aceris* HPDA25 was isolated and found to be able to promote the establishment of *G. elata-Armillaria* symbiosis.^20^ It is thereby considered an important component of *G. elata-Armillaria* symbiosis. Here, *G. elata*, *A. gallica* and *R. aceris* HPDA25 provide an opportunity to take a glimpse into the role of VOCs in *G. elata-Armillaria*-rhizosphere bacteria tripartite interactions with a simplified system.

In this study, we 1) investigated different VOCs induced by the *R. aceris* HPDA25 between non-symbiotic and symbiotic *G. elata*, and select representative VOCs in symbiotic *G. elata*; 2) explored the effects of representative VOCs on the growth of *R. aceris* HPDA25; and 3) examine the impact of representative VOCs on the growth and extracellular enzyme activity of *A. gallica*. Our results revealed for the first time of the VOC profiles of mycoheterotrophic orchids, and provided insights into the roles of VOCs in the symbiotic process between mycoheterotrophic orchids and fungi, showing that mycoheterotrophic orchids regulate the growth of symbiotic bacteria and fungi through the release of VOCs.

## Materials and methods

### Orchid, orchid mycorrhizal (OM) fungus, and mycorrhizosphere bacteria

*G*. *elata* tubers were collected from the Yunnan Province, China. The OM fungus *A. gallica* was originally isolated from the wood of cultivated *G. elata*^20^ and cultured on potato dextrose agar (PDA) medium in the dark at 25°C for 10 days. *Rahnella aceris* HPDA25 was isolated from fresh tubers^20^ and cultured in liquid Luria-Bertani (LB) medium to an optical density at a wavelength of 600 nm of approximately 0.5 at 200 rpm at 28°C.

### Co-culture of *G. elata* tubers with *A. gallica*

**Figure 1.**
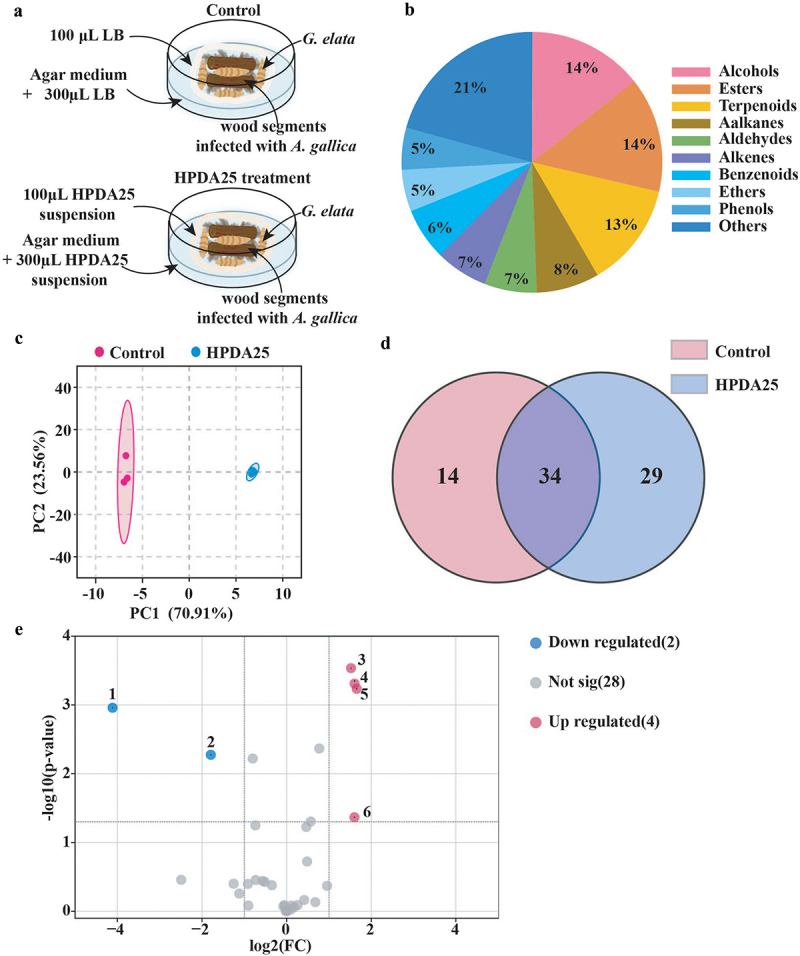
VOCs’ profile of *G. elata* with or without HPDA25-treatment.

The co-culture of immature tubers with *A. gallica* was performed in a 150 mm diameter Petri dish with treatment and control groups. A total of 30 mL of 1% agar medium was added to the Petri dishes, along with 300 μL of HPDA25 suspension as the treatment group and 300 μL of LB solution as the control group. Two 5-cm-long wood segments infected with *A. gallica* were placed on the agar medium. Subsequently, four immature tubers were gently washed with water to remove attached soil particles and soil debris. They were then surface sterilized in 75% ethanol for 30 s, soaked in 2% NaClO for 15 min, and washed three times to remove NaClO. The disinfected tubers were placed adjacent to the wood segments. In the treatment group, an additional 100 μL of HPDA25 suspension was applied around the tubers and wood segments, while the control group received an additional 100 μL of LB solution. The newly grown *G. elata* tubers were weighed after being cultured in darkness at 25°C for 90 days (Table S1). The experimental setup for the co-culture of *G. elata* tubers with *A. gallica* is shown in Figure 1a.

(a), the experimental setup for the co-culture of *G. elata* tubers with *A. gallica* and HPDA25; (b), Classification of the 77 VOCs detected by GC-MS; (c), PCA result of VOCs in the control and HPDA25-treated tubers; (d), Venn diagram of VOCs in *G. elata* tubers treated with and without HPDA25; (e), Volcano Plot of 34 common VOCs present in HPDA25-treated *G. elata* tubers and control group tubers. 1: p-Cresol. 2: Methyl rhodinolate. 3: 1,4-Dichloro-2-methoxybenzene. 4: Neryl formate/1-Bromo-3,7-dimethyl-2,6-octadiene. 5: 1-Isopropyl-2-methoxy-4-methylbenzene. 6: 1,4-Dichlorobenzene. Statistical analysis was performed using Student’s t-test (*p* < 0.05; fold change (FC) > 2).

### Measurement of VOCs from *G. elata* tubers

VOCs in *G. elata* tubers were identified using an untargeted headspace analysis through solid-phase microextraction (SPME) combined with gas chromatography-mass spectrometry (GC-MS). Fresh *G. elata* tubers were homogenized for 1 min at 30 Hz using an AM100S high-pass vibration ball mill (Ants Scientific Instruments (Beijing) Co., Ltd., China). One gram of homogenized *G. elata* tubers was transferred to a 22-mL glass headspace vial (Agilent Technologies, Inc., U.S.A.). Five microliters of 2-nonanone (0.205 μg·μL^−1^) were added as an internal standard (IS) and incubated at 30°C for 20 min. The VOCs were adsorbed onto divinylbenzene/carboxen/polydimethylsiloxane (50/30 µm) stabilized flexible fibers for 40 min, then the fibers were removed and inserted into the injector for GC-MS detection for 3 min, repeated three times.

An Agilent 7890B GC with a 7200-quadrupole time-of-flight (Q-TOF) mass spectrometer was used for GC-MS analysis. The VOCs were separated on an HP-5 MS capillary column (30 m × 0.25 mm, 0.25 μm; Agilent Technologies, Inc., U.S.A.). The flow rate was 1.5 mL·min^−1^ of He (99.999%). In the splitless mode, the injection temperature was 250°C. The initial column temperature was 50°C, rising at 2.5°C·min^−1^ to 140°C, and then at 10°C·min^−1^ to a final temperature of 230°C, which was maintained for 2 min. Electron impact ionization was performed at 70 eV with an ion source temperature at 150°C. The MS scan range was 45–650 atomic mass unit in the full-scan mode.

### Assessment of VOCs on HPDA25 growth

After HPDA25 treatment, the concentrations of 33 VOCs increased, of which 21 VOCs were accessible. The effect of 21 VOCs (Table S2) on the growth of HPDA25 was assessed in partitioned Petri dishes (90 mm diameter) by plate count method. One side of the Petri dishes was filled with LB medium and 50 µL of HPDA25 dilutions of 10^−6^ were spread plated. On the opposing side, sterile filter paper (30 mm in diameter) was placed and 100 µL of VOCs dissolved in dimethyl sulfoxide (DMSO) at varying concentrations (0.02 μg/μL, 0.2 μg/μL, 2 μg/μL, and 20 μg/μL, based on a modified protocol from Xie et al.^21^) was added. Petri dishes were immediately sealed with Parafilm ® and incubated at 30°C for 24 h, with six replicates of each group. The final colony forming units (CFU) were obtained to determine the effect of VOCs on HPDA25 growth.

### Assessment of VOCs on the growth and extracellular enzyme activity of *A. gallica*

The effect of VOCs (Table S2) on the growth of *A. gallica* was also evaluated in partitioned Petri dishes (90 mm diameter). One side of the Petri dishes was filled with PDA medium and placed with approximately 1 cm of *A. gallica* rhizomorph. On the opposing side, sterile filter paper (30 mm in diameter) was placed and 100 µL of VOCs dissolved in dimethyl sulfoxide (DMSO) at varying concentrations (0.02 μg/μL, 0.2 μg/μL, 2 μg/μL, and 20 μg/μL, based on a modified protocol from Xie et al.^21^) was added. Petri dishes were immediately sealed with Parafilm ® and incubated in the darkness at 25°C for 7 days, with six replicates of each group. The effect of VOCs on the growth of *A. gallica* was determined based on the weight and branch numbers of *A. gallica*.

To measure the extracellular enzymes activity of *A. gallica*, the PDA medium surrounding the rhizomorph was collected and centrifuged at 15,000 rpm for 15 min (4°C). Then, 500 µL of the supernatant was diluted to a total volume of 5 mL and used as an enzyme solution to measure the enzyme activity following the kit instructions (Beijing Solarbio Science & Technology Co., Ltd., China). Laccase Assay Kit for laccase activity analysis. α-Amylase (α-AL) Activity Assay Kit for amylase activity. Cellulase (CL) Activity Assay Kit for cellulase activity. Pectinase Activity Assay Kit for pectinase activity. Neutral Xylanase（NEX）Activity Assay Kit for xylanase activity. Six replicates were performed for the extracellular enzyme activity measurement.

### Transcriptome analysis of HPDA25-treated G. elata tubers

The tubers stored at −80°C were ground into powder for transcriptome analysis. Total ribonucleic acid (RNA) with three replicates of each group was extracted using RNAprep Pure Plant Plus Kit (Polysaccharides & Polyphenolics-rich, Tiangen Biotech Co., Ltd., China) according to the manufacturer’s protocol. RNA purity and quantification were evaluated using a NanoDrop 2000 spectrophotometer (Thermo Fisher Scientific Inc., U.S.A.). RNA integrity was evaluated using an Agilent 2100 Bioanalyzer (Agilent Technologies, Inc., U.S.A.). Sequencing libraries were constructed using the TruSeq Stranded messenger RNA LT Sample Prep Kit (Illumina Inc., U.S.A.). The libraries were sequenced on an Illumina sequencing platform (Illumina HiSeq X Ten, Illumina Inc., U.S.A.) and 150 bp paired-end reads were generated.

After filtering out reads containing poly-N and low-quality reads, clean reads were mapped to *G. elata* genome GWHBHOU00000000 [Genome Warehouse, China, https://ngdc.cncb.ac.cn/gwh/] using HISAT2.^22^ The read counts for each gene were obtained by HTSeq-count, and the Fragments Per Kilobase of transcript per Million mapped reads (FPKM) for each gene was calculated using Cufflinks.^23^ Differential expression analysis was performed for HPDA25-treated and control groups using the DESeq2 R package.^24^ A p-value of less than 0.05 and a log2 fold change greater than 2 were established as criteria for identifying differentially expressed genes (DEGs). Functional annotations of DEGs were performed using Gene Ontology (GO) and Kyoto Encyclopedia of Genes and Genomes (KEGG) databases with the clusterProfiler R package and visualized using Bioinformatics, a free online platform for data analysis (https://www.bioinformatics.com.cn).

### Analysis of gene expression using the quantitative real-time polymerase chain reaction (qRT-pcr)

To verify the reliability of the RNA sequencing (RNA-seq) data, 18 genes were randomly selected for real-time quantitative PCR (qRT-PCR) analysis. Total RNA from the Petri dishes was isolated using the RNAprep Pure Plant Plus Kit (Polysaccharides & Polyphenolics-rich, Tiangen Biotech Co., Ltd., China). Complementary DNA (cDNA) was synthesized using the TransScript II First-Strand cDNA Synthesis SuperMix Kit (TransGen Biotech Co., Ltd., China). The 10-fold dilution of synthesized cDNA was used as a template for qRT-PCR. Tip Green quantitative (q)PCR SuperMix Kit (TransGen Biotech Co., Ltd., China) was used for qRT-PCR with 20 μl of the mixture according to the following procedures: 94°C for 30 s, followed by 40 cycles of 94°C for 5 s, 55°C for 15 s, and 72°C for 10 s.

At the end of each qRT-PCR, a melting curve was generated using the default parameters of a LightCycler 480 II instrument (Roche Applied Science, Germany) to confirm unique amplification. All qRT-PCRs were performed in triplicate. Statistical analysis was performed using the 2^−∆∆Ct^ method. The qRT-PCR primers listed in Table S3 were designed using Primer3Plus (https://www.primer3plus.com). The elongation factor (EF)-1alpha gene was used as an internal reference.^25^

### Identification of key genes involved in the biosynthesis of VOCs

VOCs are categorized into terpenes, benzenoids, and fatty acid derivatives. The key biosynthetic genes associated with these compounds include acetaldehyde dehydrogenase (*ALDH*), terpene synthase (*TPS*), cytochrome P450 monooxygenases (*CYP*), short-chain dehydrogenase (*SDR*), carotenoid cleavage dioxygenase (*CCD*), double bond reductase (*DBR*), chorismate mutase (*CM*), phenylalanine ammonia lyase (*PAL*), lipoxygenase (*LOX*) and hydroperoxide lyase (*HPL*).^26–28^ The key symbiosis-related genes include subtilisin-like protease (SBT) and pectin methylesterase (PME).

The *Arabidopsis thaliana* protein sequences of the above genes were downloaded from The Arabidopsis Information Resource (https://www.arabidopsis.org/). The CYP71D-subfamily, CYP76S-subfamily, CYP736A-subfamily genes from *Mentha spicata, Mentha x piperita, Perilla frutescens, Thymus vulgaris, Origanum vulgare, Sorbus aucuparia, Malus domestica, Bupleurum chinense* and *Origanum vulgare* were downloaded from National Center for Biotechnology Information (NCBI) according to the accession numbers (Table S4). The *CCD10* genes from *Nicotiana tabacum*, *DBR* genes from *Artemisia annua*, and *HPL* genes from *Oryza characterize* and *Medicago trunculata* were downloaded from NCBI according to the accession numbers (Table S4). Hidden Markov model (HMM) profiles were obtained using InterPro (https://www.ebi.ac.uk/interpro/). HMM searches were used to identify all possible genes using TBtools software. Genes without Pfam numbers were identified using BLASTP (e-value <1e^−5^). InterProScan (https://www.ebi.ac.uk/interpro/.search/sequence/) and NCBI Conserved Domains Database (CDD) (https://www.ncbi.nlm.nih.gov/cdd/) were used to determine the corresponding conserved domains of the reference genes. Multiple sequences of reference proteins from *G. elata* and reference species were aligned using MUSCLE with default parameters to construct a phylogenetic tree using the neighbor-joining method with 1,000 bootstrap replicates via MegaX. All corresponding protein accession numbers are listed in Table S4.

### Statistical analysis

Data were statistically analyzed using t-tests in SPSS ver20.0. For the GC-MS experiment, the VOCs were identified by comparing the retention time and mass spectra in combination with those of authentic compounds or with those of the National Institute of Standards and Technology (NIST).^29^ Quantitative analysis was performed by comparing peak areas with those of the IS. The calculation formula is as follows: component content (ng·g^−1^) = [(component peak area/IS peak area) × IS concentration (μg·μL^−1^) × IS volume (μL)] × 1,000/sample weight (g).

Multivariate analyses were performed using the MetaboAnalyst 6.0 (https://www.metaboanalyst.ca). Data were normalized to the median and transformed using a generalized logarithmic scheme. Principal component analyses (PCA) were performed according to Pareto correlation.

Differences between control and treatment groups were analyzed with the Dunnett’s test.

## Results

### HPDA25 induced VOCs change in *G. elata* tubers

VOC profiles of HPDA25-treated and untreated *G. elata* tubers were analyzed. A total of 77 VOCs were detected, with 71 identified. These compounds comprised 11 alcohols and esters, 10 terpenoids, 6 alkanes, 5 aldehydes, 5 alkenes, and 5 benzenoids, along with 4 ethers and phenols (Table S5). Among these classifications, alcohols, esters and terpenoids accounted for the greatest number of VOCs (Figure 1(b)). Furthermore, concentrations of 27 VOCs were found to be over 10 ng·g^−1^ (Table 1).

**Table 1.** VOCs with concentrations over 10 ng · g^−1^ (ng · g^−1^, *n* = 3, mean ± SD).

	Chemspider name	CAS	HPDA25	Control
1	Styrene	100-42-5	1591.23 ± 359.03	2186.31 ± 1572.43
2	Unknown-1	–	463.35 ± 94.62	986.94 ± 881.13
3	p-Cresol	106-44-5	37.40 ± 17.87**	719.16 ± 441.73
4	1-Hexanol	111-27-3	540.63 ± 24.45	643.76 ± 462.6
5	2-Pentylfuran	3777-69-3	548.33 ± 11.35	397.18 ± 101.72
6	Methyl hexoate	106-70-7	152.40 ± 9.95	92.44 ± 33.05
7	Oxirane, 2-(1,1-dimethylethyl)-3-ethyl-, cis-	36099-44-2	138.98 ± 8.76	110.50 ± 91.83
8	Unknown-4	–	33.05 ± 4.52	67.10 ± 63.01
9	3-Octanone	106-68-3	55.73 ± 11.48	38.7 ± 5.02
10	2-Octanone	111-13-7	–	44.08 ± 22.91
11	1-Octen-3-ol	3391-86-4	28.78 ± 1.61	33.73 ± 21.67
12	3-Methylpentane	96-14-0	–	29.81 ± 24.44
13	1-Isopropyl-2-methoxy-4-methylbenzene	1076-56-8	27.98 ± 2.24**	9.52 ± 3.01
14	2-Heptanone	110-43-0	27.23 ± 6.31	–
15	Caprylic acid methyl ester	111-11-5	19.58 ± 0.9	26.82 ± 14.03
16	2-Hexyloxirane	2984-50-1	22.31 ± 2.89	–
17	Unknown-3	–	–	20.25 ± 19.57
18	3-Methyl-1-heptene	4810-09-7	18.33 ± 2.48	–
19	Hexanal	66-25-1	17.56 ± 3.67	–
20	2-ethylcyclohexan-1-ol	3760-20-1	15.01 ± 2.12	16.15 ± 11.35
21	1,3-Di-tert-butylbenzene	1014-60-4	10.06 ± 6.15	14.42 ± 1.84
22	1-Heptanol	111-70-6	9.19 ± 0.95	14.71 ± 10.28
23	Unknown-2	–	13.04 ± 0.79	–
24	Methyl valerate	624-24-8	12.30 ± 1.94	–
25	1,4-Dichlorobenzene	106-46-7	10.72 ± 3.38*	3.38 ± 1.32
26	Sylvestrene	1461-27-4	10.61 ± 3.05	10.86 ± 3.51
27	2-Methylcyclohexanol	583-59-5	10.62 ± 1.22	–

Compared with control group, *p < 0.05, **p < 0.01.

To evaluate HPDA25-mediated modulation of VOCs in *G. elata* tubers, VOC numbers and concentration changes were visualized using Venn diagram and volcano plot, respectively. PCA analysis showed a distinct separation of VOCs between *G. elata* tubers treated with or without the strain HPDA25 (Figure 1(c)). The VOCs of each group were separated by PC1 and PC2, where PC1 and PC2 represented 70.91% and 23.56% of the variation, respectively. The Venn diagram showed that *G. elata* tubers treated with HPDA25 had a higher number of VOCs, with 63 VOCs identified, compared to 48 VOCs identified in untreated tubers. Of these, 29 VOCs were only found in tubers treated with HPDA25, whereas 14 VOCs were unique to the tubers in the control group (Figure 1(d)). For the remaining 34 VOCs present in both groups, concentrations of 4 VOCs were significantly higher in the HPDA25-treated group, whereas concentrations of 2 VOCs were significantly higher in the control group (Figure 1(e)). Overall, HPDA25 increased the concentrations of a total of 33 VOCs.

### Only hexanal showed antagonistic effect on the growth of HPDA25

**Figure 2.**
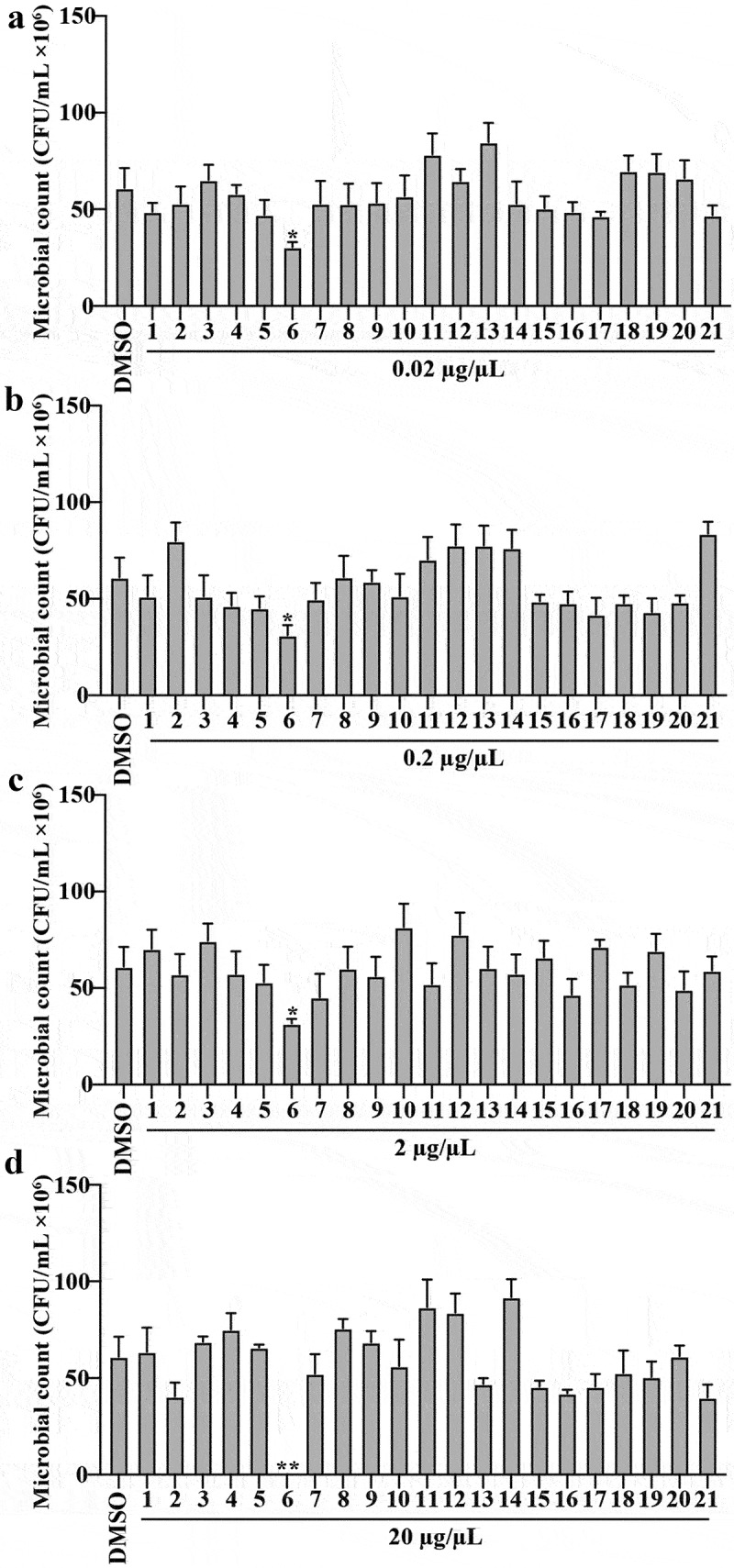
Effect of the VOCs with significantly increased levels on the growth of HPDA25.

To evaluate the effect of VOCs on HPDA25 growth, 21 commercially available VOCs with significantly increased concentrations by HPDA25 (Table S2) in *G. elata* tubers were co-cultured with HPDA25. As shown in Figure 2, only hexanal inhibited the growth of HPDA25 at all concentrations tested, with other VOCs showing no significant effect on HPDA25 growth (Table S6). These results indicated that HPDA25 facilitated VOC emission in *G. elata*, while the VOCs could not promote the growth of HPDA25.

(a): The numbers of HPDA25 subjected to VOCs at 0.02 µg/µL; (b): The numbers of HPDA25 subjected to VOCs at 0.2 µg/µL; (c): The numbers of HPDA25 subjected to VOCs at 2 µg/µL; (d): The numbers of HPDA25 subjected to VOCs at 20 µg/µL. 1, dihydro-beta-ionone; 2, nerol; 3, decanal; 4, butylated hydroxytoluene; 5, thymoquinone; 6, hexanal; 7, 2-methylcyclohexanol; 8, benzaldehyde; 9, methyl valerate; 10, phenylethyl alcohol; 11, 2-methylisoborneol; 12, 1,4-dichloro-2-methoxybenzene; 13, benzeneacetaldehyde; 14, 2-heptanone; 15, 1-isopropyl-2-methoxy-4-methylbenzene; 16, 2-ethylhexanol; 17, duroquinone; 18, chloroneb; 19, 4-(1-methylpropyl)-phenol; 20, 2-hexyloxirane; 21, 2,4-dimethyl-1-heptene. Values are presented as mean ± SE (*n* = 6). Asterisks indicate statistically significant differences compared to DMSO group as determined by Dunnett’s test: **p < 0.01.

### Six elevated VOCs in symbiotic G. elata inhibited the growth of *A. gallica*

**Figure 3.**
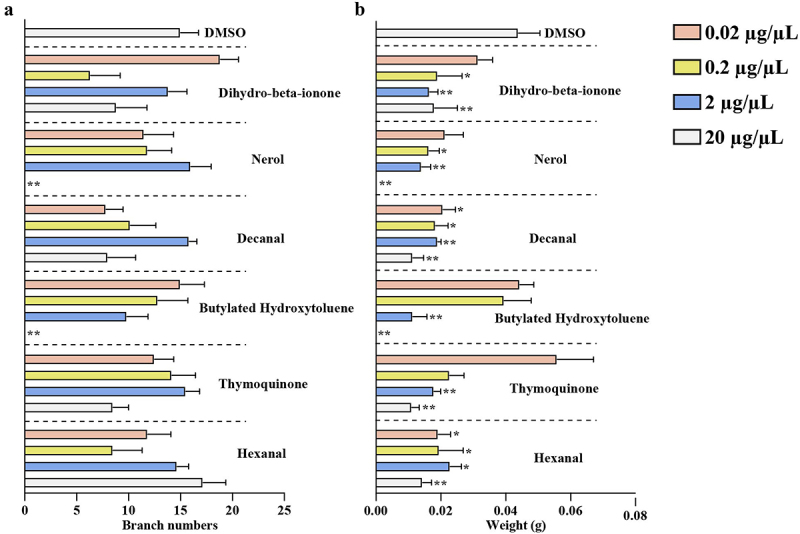
The six VOCs with significantly elevated concentrations inhibited the growth of *A. gallica*.

The absence of any notable impact on HPDA25 by volatiles suggests that these VOCs might potentially influence *A. gallica* growth, the main participant in the *G. elata-A. gallica* symbiotic relationship. To assess the impact of VOCs on the growth of *A. gallica*, 21 commercially available VOCs showing significantly elevated levels (Table S2) were examined for their effects on the growth of *A. gallica* rhizomorphs. As solvent, DMSO did not affect the growth of *A. gallica* (Table S7). Decanal and hexanal obviously reduced the rhizomorph weight of *A. gallica* at all concentrations, resulting in a reduction ranging from 0.60 to 0.81-fold. Nerol and dihydro-beta-ionone exhibited significant suppression of rhizomorph weight at concentrations of 0.2, 2, and 20 µg/µL, resulting in a reduction of 0.44 to 0.75-fold. Thymoquinone and butylhydroxytoluene had inhibitory effects on rhizomorph weight only at concentrations of 2 and 20 µg/µL. The lack of *A. gallica* growth indicated that the growth of *A. gallica* was completely inhibited by treatment with nerol and butylhydroxytoluene at 20 µg/µL (Table S7 and Figure 3(a-b)). Other compounds did not affect its growth (Table S7). Six compounds inhibited the growth of *A. gallica*, indicating that HPDA25 induced an increase in VOC concentrations in *G. elata*, which could enhance its antifungal effect on *A. gallica*.

(a): effect on *A. gallica* branch numbers; (b): effect on *A. gallica* weight. Values are presented as mean ± SE (*n* = 6). Asterisks indicate statistically significant differences compared to DMSO group as determined by Dunnett’s test: *p < 0.05 and **p < 0.01.

### Six elevated VOCs in symbiotic G. elata reduced extracellular enzyme activities of *A. gallica*

**Figure 4.**
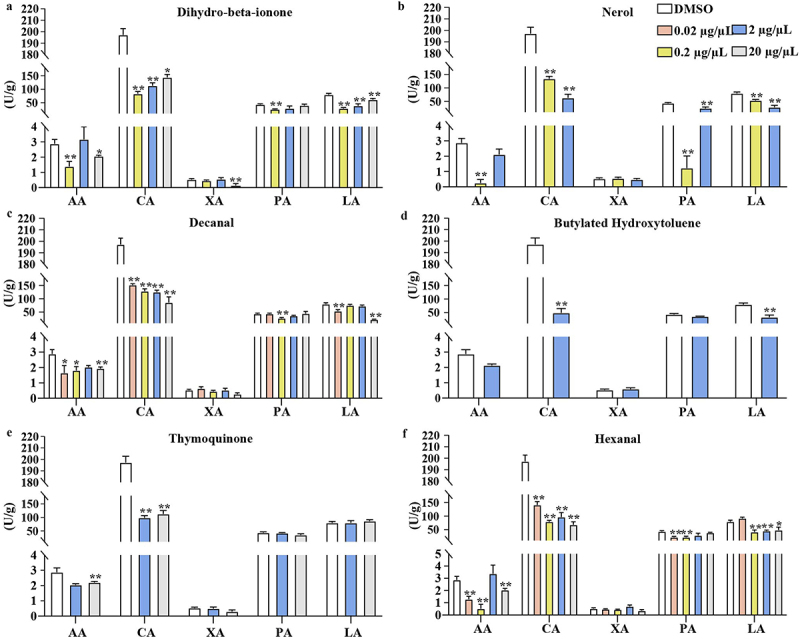
The impact of six VOCs on the activity of extracellular enzymes.

The growth of *A. gallica* is closely associated with extracellular enzyme activity which can degrade plant cell walls for nutrient uptake.^20^ The inhibition of *A. gallica* growth by six active VOCs indicates a potential impact on extracellular enzyme activities. As solvent, DMSO showed no effect on the extracellular enzyme activities of *A. gallica* (Table S8). As shown in Figure 4, dihydro-beta-ionone significantly decreased cellulase and laccase activity at 0.2, 2, and 20 µg/µL from 0.22 to 0.63-fold. Dihydro-beta-ionone significantly decreased amylase activity, xylanase activity and pectinase activity at 0.2 and 20 µg/µL from 0.10 to 0.99-fold.

(a) dihydro-beta-ionone, (b) nerol, (c) decanal, (d) butylated hydroxytoluene, (e) thymoquinone, and (f) hexanal. AA: Amylase activity; CA: Cellulase activity; XA: Xylanase activity; PA: Pectinase activity; LA: Laccase activity. Values are presented as mean ± SE (*n* = 6). Asterisks indicate statistically significant differences compared to DMSO group as determined by Dunnett’s test: *p < 0.05 and **p < 0.01.

Nerol caused a substantial reduction in cellulase activity and laccase activity at concentrations of 0.02, 0.2, and 20 µg/µL, resulting in a drop of 0.31 to 0.68-fold. Nerol caused a considerable reduction in amylase activity at concentrations of 0.2 µg/µL, reducing it by 0.94-fold, respectively. Nerol exhibited a substantial reduction in pectinase activity at concentrations of 0.2 and 2 µg/µL, resulting in a drop of 0.97-fold and 0.42-fold, respectively. However, it did not inhibit xylanase activity.

Decanal exhibited a considerable reduction in cellulase activity across all tested doses, ranging from 0.23 to 0.56-fold drop. Decanal exhibited a considerable reduction in pectinase activity at concentrations of 0.2 µg/µL, a drop-in laccase activity at concentrations of 0.02 and 20 µg/µL, as well as drop-in amylase activity at 0.02, 0.2 and 20 µg/µL. The reduction in pectinase, laccase and amylase activity ranged from 0.33 to 0.97-fold. However, it did not inhibit xylanase activity.

At a concentration of 2 µg/µL, butylated hydroxytoluene caused a considerable drop in cellulase and laccase activity, ranging from 0.58 to 0.75-fold. However, amylase, pectinase and xylanase activity were not affected. Thymoquinone exhibited a substantial reduction in amylase activity at concentrations of 20 µg/µL, resulting in a drop of 0.99-fold. Thymoquinone significantly decreased cellulase activity at 2 and 20 µg/µL, by 0.50 and 0.42-fold, respectively. Thymoquinone did not decrease xylanase, pectinase and laccase activity.

Hexanal had a strong inhibitory effect on cellulase activity at all tested doses, reducing it by 0.28 to 0.65-fold. Hexanal caused a considerable reduction in amylase activity at concentrations of 0.02, 0.2, and 20 µg/µL, reducing it by a factor of 0.55 to 0.99. Hexanal caused a considerable reduction in pectinase activity at concentrations of 0.02 and 0.2 µg/µL, resulting in a drop of 0.47 to 0.48-fold. Hexanal caused a substantial drop-in laccase activity at concentrations of 0.2, 2, and 20 µg/µL. The laccase activity was reduced to 0.39 to 0.47 times its original level. However, xylanase activity was not affected (Table S8 and Figure 4).

### Symbiosis elevates transcript levels of key genes in the biosynthesis of six active VOCs

**Figure 5.**
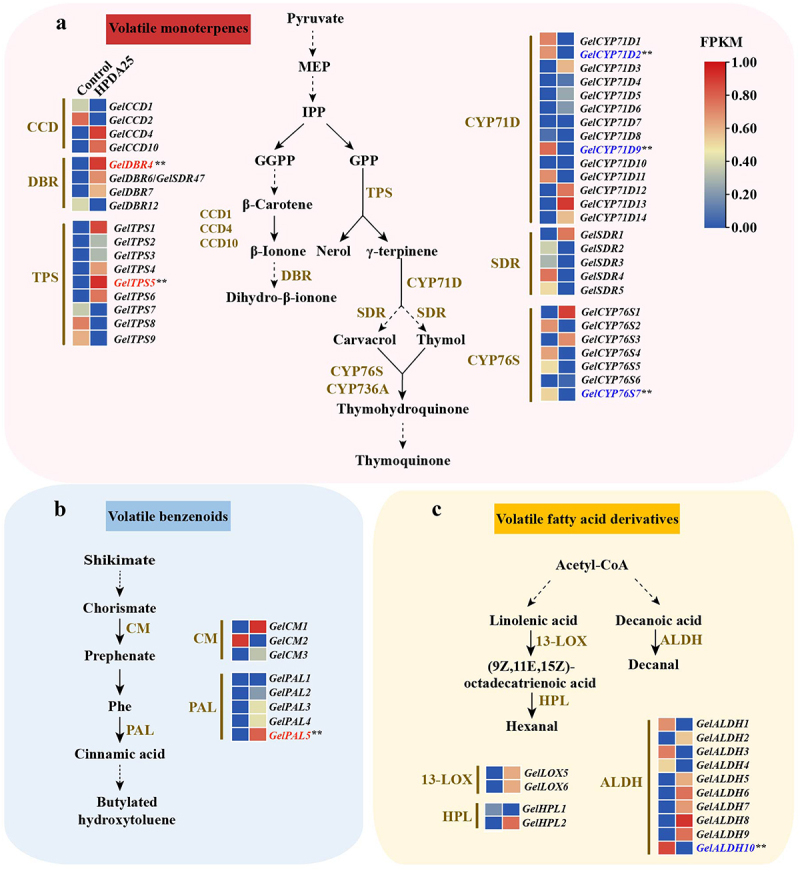
Scheme of VOCs biosynthesis.

In order to further investigate whether the transcript levels of genes in the VOC biosynthesis pathway are affected by the symbiotic process, the expression profile of the symbiotic *G. elata* was characterized using RNA-seq and changes in the transcript levels of seven genes in six active VOCs biosynthesis were investigated using qRT-PCR, RNA-seq and qRT-PCR and results were consistent (Table S9),

Among the six active VOCs, nerol, dihydro-beta-ionone and thymoquinone are volatile monoterpenes; butylated hydroxytoluene is benzenes; and decanal and hexanal are derived from fatty acid.^30^ Volatile monoterpenes are generated from methylerythritol phosphate (MEP) pathway^26^ (Figure 5a). In the pathway, isopentenyl diphosphate (IPP) is converted to geranylgeranyl diphosphate (GGPP) and geranyl diphosphate (GPP). Then, GGPP is converted to β-carotene, which is then cleaved into β-ionone by CCD1, CCD4 and CCD10.^31–33^ There is no apparent difference in the expression pattern of four *G. elata* genes analogous to CCD1, CCD4 and CCD10 (Table S4 and Table S9). DBR1 has been proved to convert β-ionone to dihydro-beta-ionone. Four homologous to AaDBR1 were identified in *G. elata* (Table S4), among which only the transcript level of *GelDBR4* was strongly increased after symbiosis (Table S9). GPP is catalyzed by TPS-b and TPS-g, two subfamilies of the TPS family, to form monoterpenes,^34^ such as nerol. In *G. elata*, nine genes (*GelTPS1-GelTPS9)* belonged to the TPS-b subfamily, with no members from the TPS-g subfamily (Table S4). Notably, only the transcript level of GelTPS5 was significantly increased following symbiosis (Table S9). The thymohydroquinone is the precursor of thymoquinone, which is sequentially synthesized from γ-terpinene through the catalysis of CYP71D, SDR, and CYP76S/CYP736A.^35^ The gene transcript levels for these three enzymes did not significantly increase (Table S9).

(a): pathway for dihydro-beta-ionone, nerol, and thymoquinone biosynthesis. (b): pathway for butylated hydroxytoluene biosynthesis. (c): pathway for hexanal and decanal biosynthesis. Upregulated DEGs were in red; downregulated DEGs were in blue; no significant genes were in black. Control, the co-culture system was untreated with HPDA25; HPDA25, the co-culture system was treated by HPDA25. MEP, methylerythritol phosphate. IPP, isopentenyl pyrophosphate. GPP, geranyl pyrophosphate. GGPP, geranylgeranyl pyrophosphate. CCD: carotenoid cleavage dioxygenase. DBR: double bond reductase. TPS, terpene synthase. CYP, cytochrome P450 monooxygenases. SDR, short-chain dehydrogenase. CM, chorismate mutase. Phe, phenylalanine. PAL, phenylalanine ammonia lyase. LOX, lipoxygenase. HPL, hydroperoxide lyase. ALDH, acetaldehyde dehydrogenase. Values are presented as mean ± SE (*n* = 3). Asterisks indicate statistically significant differences compared to control group as determined by Student’s t-test: *p < 0.05 and **p < 0.01.

Butylated hydroxytoluene is initiated from shikimate, which is catalyzed into chorismate and subsequently converted into prephenate by CM^28^ (Figure 5b). Phenylalanine (Phe) from prephenate is further deaminated to cinnamic acid by PAL and further synthesizes butylated hydroxytoluene.^26^ Three *CM* genes and 5 *PAL* genes were identified, of which one PAL gene (*GelPAL5*) were highly induced after HPDA25 treatment while CM genes (*GelCM1-GelCM3*) showed no significant differences (Table S9).

Biosynthesis of fatty acids starts from acetyl-CoA^36,37^ (Figure 5(c)). A variety of volatile fatty acid derivatives are synthesized from C_18_ unsaturated fatty acids which were transformed to (9Z,11E,15Z)-octadecatrienoic acid (13-HPOD) by 13-LOX^38^ (Figure 5(c)). 13-HPOD is converted to hexanal by 13-HPL.^26^ Two *LOX* genes belong to 13-LOX were identified and showed no differential expression. Two *HPL* genes (*GelHPL1, GelHPL2*) in LOX pathway were identified and showed no differential expression after symbiosis (Table S9). Decanoic acid is the substrate for producing decanal by ALDH enzyme.^39^ Ten ALDH genes were identified, of which the transcript level of *GelALDH10* was down regulated by HPDA25 (Table S9).

Overall, the transcript level of three genes involved in the biosynthesis of six active VOCs was increased after symbiosis (Figure 5), thereby leading to the hypothesis that these genes as key contributors to the promotion of the VOCs synthesis.

### HPDA25 promoted the expression of symbiosis-related genes

In our earlier investigation,^20^ it was shown that HPDA25 stimulated the development of *G. elata*. A comparative transcriptome study was conducted on tubers treated with or without HPDA25 to gain a deeper understanding of the underlying processes in *G. elata*‘s response to HPDA25. A total of 2,285 DEGs were identified, including 920 up-regulated and 1,365 down-regulated expressed genes.

**Figure 6.**
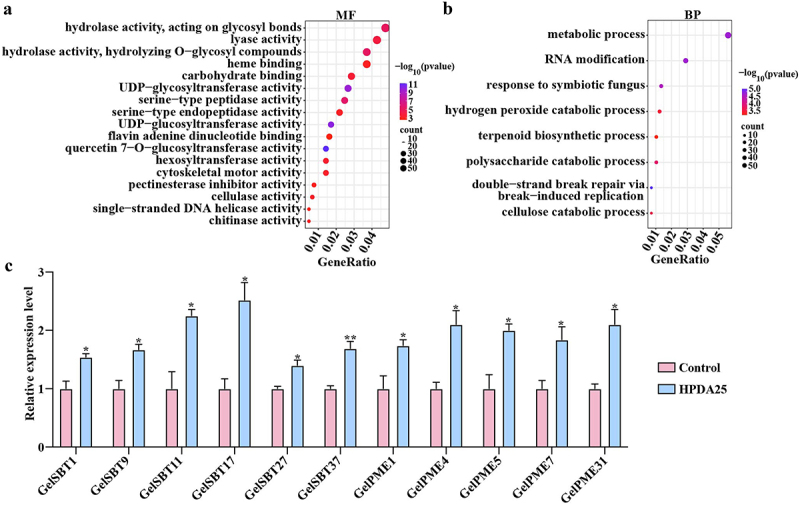
Enrichment analysis of DEGs.

We explored in detail the genes on each of the enriched pathways (Table S10 and Table S11). The study focused on pathways associated with symbiosis, specifically examining molecular functions (MF) and biological processes (BP) linked to cellulase activity, pectinesterase inhibitor activity, and response to symbiotic fungus (Figure 6(a-b)). Gene expression analysis showed that 11 genes encoding subtilisin-like proteases (*GelSBT1, GelSBT9, GelSBT11, GelSBT17, GelSBT27, GelSBT37*) and pectin methylesterases (*GelPME1, GelPME4, GelPME5, GelPME7, GelPME31*) were upregulated (Figure 6(c)).

(a): MF, (b): BP, (c): the relative transcript levels of *SBT* and *PME* gene Values are presented as mean ± SE (*n* = 3). Asterisks indicate statistically significant differences compared to control group as determined by Student’s t-test: *p < 0.05 and **p < 0.01.

**Figure 7.**
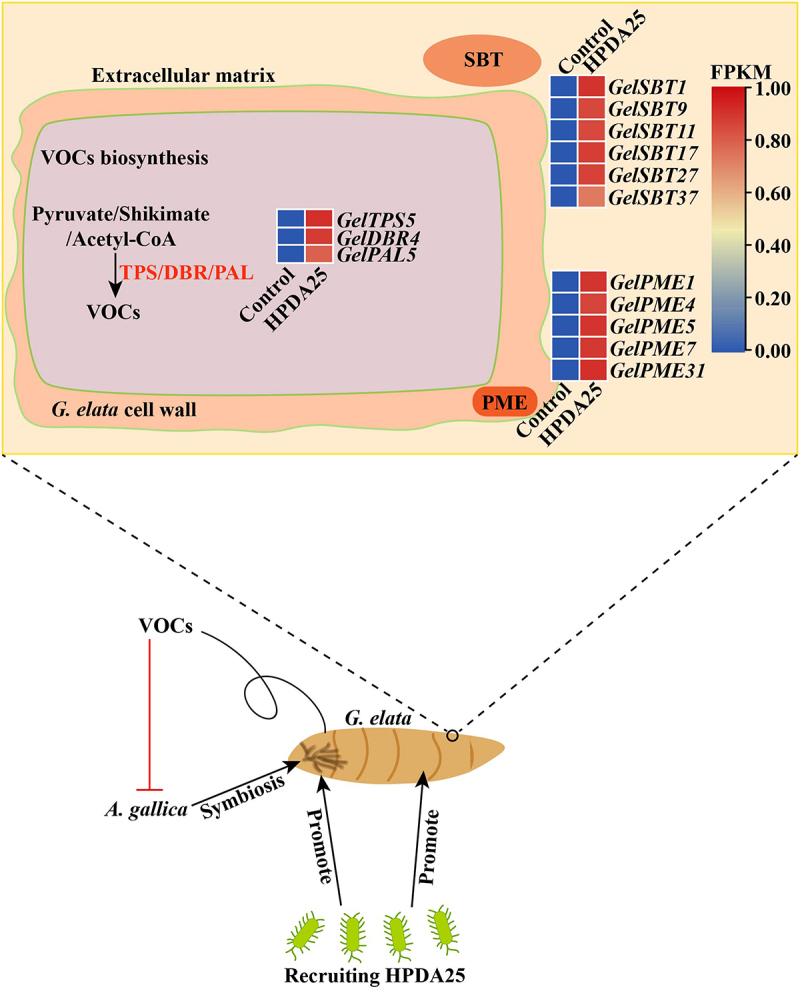
A graphical illustration of the balancing role of VOCs in the *G. elata-Armillaria* symbiotic relationship using HPDA25 as a promotor.

These results showed that PGPB HPDA25 which promote the growth of *G. elata* and *A. gallica* promoted more VOCs production in *G. elata*. VOCs inhibited the growth and extracellular enzyme activity of *A. gallica* and showed as balancer to regulate the *G. elata-Armillaria* symbiosis process. HPDA25 enhanced the activity of VOC biosynthesis and symbiosis-related genes, aiding *G. elata* in resisting *A. gallica*. SBTs are dispersed within the extracellular matrix of plants and exhibit defensive properties in response to encounters with pathogens. PMEs enhance the stiffness of the plant cell wall to inhibit the infiltration of pathogens Figure 7.

## Discussion

Plants synthesize a diversity of VOCs to communicate with other organisms, including bacteria and fungi.^40^ However, the effect of VOCs on the *G. elata-Armillaria* symbiosis remains unclear. This study reveals that the profile of VOCs in HPDA25-inducible symbiotic *G. elata* differs from that of non-symbiotic *G. elata*. This finding strongly suggests that VOCs are actually involved in the symbiotic process. One challenge is determining whether these differential VOCs are a result of symbiosis or are directly caused by HPDA25. Recent advances have demonstrated that beneficial microbes can induce VOC release in plants.^41^ Treatment with plant growth-promoting rhizobacteria (PGPR) induces the production of volatiles such as indole,^42^ β-caryophyllene,^43^ and volatile terpenes^44^ that affect defense responses. The identified VOCs in *G. elata* promoted by HPDA25, such as 1,4-dichloro-2-methoxybenzene, neryl formate, 1-isopropyl-2-methoxy-4-methylbenzene, and 1,4-dichlorobenzene, have not been previously documented in VOCs induced by plant growth-promoting rhizobacteria (PGPR) in autotrophic plants. Thus, these molecules are assumed to be connected to symbiosis and were chosen as representative VOCs. Among the 33 representative VOCs from *G. elata*, 21 were available for investigation of their effects on HPDA25 and *A. gallica*, the other two participants in the *G. elata-A. gallica*-HPDA25 tripartite system. Previous studies on VOCs induced by PGPR in plant–microbe interactions have primarily focused on their inhibitory effects on pathogenic fungi,^43,45^ often neglecting their effects on the PGPR itself. To date, only γ-caprolactone, γ-decalactone, and γ-nonalactone have demonstrated a positive effect in attracting beneficial bacteria from bulk soil.^46^ Our results indicated that, out of these 21 VOCs, only hexanal exhibited an inhibitory effect on HPDA25 growth, with no compounds showing any promoting activity. These findings indicate that other types of substances, such as *G. elata* root exudates, may play critical roles in regulating bacteria in *G. elata* symbiotic system. Therefore, future research on substances other than VOCs is highly necessary.

The effects of representative VOCs on *A. gallica* are of greater concern, as *Armillaria* play a more critical role in this symbiotic system.^2^ Among 21 tested representative VOCs, six were capable of inhibiting *A. gallica* growth, while none promoted. Of these six compounds, two are aldehydes, three are terpenes, and the remaining is benzene. Except for dihydro-beta-ionone, all other compounds have been reported to exhibit antifungal activity, including nerol,^47^ thymoquinone,^48^ decanal,^49^ hexanal^50^ and butylated hydroxytoluene.^51^ *Armillaria* species secrete a series of extracellular enzymes to degrade plant cell wall for nutrition and colonization.^20^ As *A. gallica* growth were inhibited by six VOCs mentioned above, the extracellular enzymes of *A. gallica* treated with the VOCs *in vitro* were examined. *Armillaria* species utilize different extracellular enzymes to degrade different plant cell wall compositions, thereby obtaining nutrition from plants.^3^ Laccase, cellulase, pectinase, xylanase and amylase activities were all inhibited by the six VOCs mentioned above. In addition to acquiring nutrients from plant cells,^52^ these enzymes also facilitate invasion of *A. gallica* into the cortex of *G. elata*.^20^ Considering the impact of VOCs on the growth and extracellular enzyme activity of *A. gallica*, these findings suggest that VOCs might play an inhibitory role in preventing *A. gallica*, invasion during *G. elata-Armillaria* symbiosis. This hypothesis is consistent with previous reports that most VOCs predominantly inhibit pathogenic fungi, with only limited evidence of VOCs benefiting fungi.^53^

Furthermore, the key genes involved in the six VOCs biosynthesis were assessed. DBR is an important enzyme for the biotransformation from β-ionone to dihydro-beta-ionone.^54^ TPS is an important enzyme for the biosynthesis of volatile terpenoids from prenyl diphosphate substrates.^26^ The elevated expressions of *GelDBR4* and *GelTPS5* pave the way for the increased concentrations of nerol and dihydro-beta-ionone. PAL is an enzyme that catalyzes phenylalanine to produce benzenoids.^28^ The transcript level of *GelPAL5* was significantly increased by HPDA25-induced symbiosis, play a key role in an elevated concentration of butylated hydroxytoluene.

Additionally, we have conducted preliminary explorations into other changes occurring in HPDA25-induced symbiotic *G. elata* and found that the transcript levels of genes coding for subtilisin-like proteases and PMEs were up-regulated. Subtilisin-like proteases are serine peptidases associated with plant defense activity during plant–pathogen interactions and located in the plant extracellular matrix.^55^ SBT3 was induced in response to wounding in injured leaves of tomato when attacked by herbivore *Manduca sexta*.^56^ PMEs have been reported roles in defense responses. An increase in PME activity promotes the rigidity of the plant cell wall and defense against pathogens.^57^ Therefore, these results suggested that HPDA25 may facilitate *G. elata* inhibit *A. gallica* as well.

## Conclusion

VOCs play a critical role in plant–microbe interactions; however, the VOCs of mycoheterotrophic plant have remained largely unexplored. This study suggests that VOCs undergo changes during the symbiotic formation process of *G. elata* and several active VOCs were identified through growth assays and extracellular enzyme assays of *Armillaria*, including nerol, thymoquinone, decanal, hexanal, dihydro-beta-ionone, and butylhydroxytoluene. Our results suggest that these VOCs inhibit *A. gallica* in the *G. elata-Armillaria* symbiosis, but have no significant effect on HPDA25, the PGPR in this system. Therefore, further research needs to be carried out to explore substances that regulate bacteria in this symbiotic system. Moreover, the VOCs identified in this study are all inhibitory, indicating there are other compounds with positive functions on *Armillaria* to maintain *G. elata-Armillaria* homeostasis.

## Supplementary Material

Supplementary tables.xlsx

## Data Availability

All transcriptomic sequencing data generated in this study are available at the NGDC Genome Sequence Archive (accession number: CRA018380; BioProject number PRJCA018493).
